# Emergency contraceptive use of Metronidazole among University female students in Dodoma region of Tanzania: a descriptive cross-sectional study

**DOI:** 10.1186/s40834-023-00241-z

**Published:** 2023-08-22

**Authors:** Nipael M. Samson, Emmanuel Izack Sumari, Valence Ndesendo, Romuald Mbwasi

**Affiliations:** 1 Department of Pharmaceutics, School of Pharmacy and Pharmaceutical Sciences, John’s University of Tanzania, Formulation Sciences, and Pharmaceutical Services, Dodoma, Tanzania; 2 Department of Nursing Management, Muhimbili University of Health and Allied Sciences, Dar Es Salaam, Tanzania

**Keywords:** Metronidazole, Emergency contraceptive, Use, Female students, Dodoma, Tanzania

## Abstract

**Background:**

Metronidazole is known for its therapeutic effect as antibacterial and anti-parasitic. However, its toxicity on the reproductive system remains unclear. Metronidazole use in rodents is associates with toxic effects on the reproductive system, including hormonal alterations, reduced number of fertile cells and reduced sites for implantation, size of the placental disc area, constituent elements of the labyrinth, and spongiotrophoblast layers. Its use at a therapeutic dose among humans has been associated with an increased risk of spontaneous abortion. The effects on the reproductive system in humans may result in misconceptions about contraceptive effects hence sexually active individuals like students who, for any reason, fail to access safe contraceptive services use any possible methods to protect them from conception. This study aims to investigate the unofficial (un-prescribed) use of Metronidazole as an emergency contraceptive and some of its associated factors.

**Methods:**

This quantitative cross-sectional study involved 470 participants where stratified random sampling technique was used to obtain the sample from three educational institutions in the Dodoma Municipal, Dodoma region. Collected data were analyzed using SPSS version 25, descriptive statistical analysis was done to determine frequencies, percentages, and association, *p* < *0.05* was used to determine statistical significance. Further analysis using Multivariate binary logistic regression was done to determine the nature of the association between the study variables.

**Results:**

The finding shows that 169(62.4%) use Metronidazole as an emergency contraceptive. Notably, 345(73.4%) stated that they had ever heard someone use Metronidazole for contraception, especially their peers. Furthermore, an increase in the year of study was significantly associated with reduced use of Metronidazole as an emergency contraceptive (B = [-0.45], *p* = [0.02]). Furthermore, an increase in age, studying in non-medical college/university, the experience of using contraceptive methods, and hearing someone ever used Metronidazole was found to be positively associated with its use as an emergency contraceptive, although not statistically significant.

**Conclusion:**

Metronidazole was found to be used as an emergency contraceptive in high doses, different factors associated with its use, and reasons influencing its use. Further research may be done to explore the toxicological effect of high doses of Metronidazole as a contraception and compare the efficiency of Metronidazole over other emergency contraceptives.

## Introduction

### Background

Metronidazole was introduced many years ago and is known for its therapeutic effect as antibacterial and anti-parasitic. However, its toxicity on different body systems remains not well studied [1, 2]. This medicine was first used in 1959 for treating infection caused by *Trichomonas vaginalis*. Further discoveries proved that the drug effectively treated gastrointestinal, cardiovascular, neurological, respiratory, dermatological, and gynecological infections [1–3]. It has also commonly been used as a prophylaxis before surgical operations, and it is made available in different forms such as tablets, solutions, and tube [1].

The mild to moderate side effects of Metronidazole can be tolerated. Still, some toxic effects such as genotoxicity, neurotoxicity, optic, and peripheral neuropathy, and encephalopathy have been reported, especially on prolonged and higher doses use [2]. Several studies have been done on mice and dogs to understand its effects on the reproductive system, showing some toxic effects, although these have not been studied much in humans [1, 2]. Results on humans exposed to the high dose of 750 mg of Metronidazole for a long time showed mutagenic activity in the urine [4]. In addition, female rats that were given Metronidazole for a long time had increased neoplasms, especially mammary tumors [2]. These results have made Metronidazole associated with carcinogenicity and mutagenicity among the serious toxic effects [2, 5].

Effects of Metronidazole on the reproductive system have also been studied in mice, rats, dogs, and little in humans. The use of Metronidazole in male mice proved that it significantly affects the male reproductive system and male fertility by reducing the number of mobile sperms following two weeks of 500 mg administration [6]. It also reduced the weight of the testes, epididymides, seminal vesicles, and prostates two months after the therapy's initiation, which was explained by reduced testosterone levels [6, 7]. In addition, Metronidazole was associated with the reduction of reproductive hormones such as follicle-stimulating hormones, luteinizing hormones, and massive degeneration of the germ cells at stage VII [7]. The effect of Metronidazole on the placenta and fetal development in rats revealed that 130 mg/kg was toxic since it reduced the number of implanted embryos, weight, and components of the placenta and neonates, suggesting cautious use in pregnancy on prolonged use [8].

The study investigating whether Metronidazole crosses the placenta revealed positive results, although its effect on the fetus is controversial [5]. A clinical study reports that following 1 h application of 1 g of metronidazole gel significant spermicidal effect was observed [9]. Furthermore, mothers who used Metronidazole during pregnancy gave birth to newborns with suprarenal neuroblastoma [5]. Another study that investigated the effects of macrolides and Metronidazole on fetal attachment showed that Metronidazole use was associated with about 70% increased risk of spontaneous abortion [10].

The few studies that have documented Metronidazole reproductive toxicity mainly focused on effects in women using the medicine to treat other conditions and not on whether they were used specifically as an emergency contraceptive or to induce abortion.

## Methodology

### Aim of the study

This study aims to investigate the unofficial (un-prescribed) use of Metronidazole as an emergency contraceptive and some of its associated factors.

### Study design

A quantitative cross-sectional study design was used, to quantify the unofficial (unprescribed) use of Metronidazole as an emergency contraceptive, and data were collected for two months, from May to July 2021.

### Study setting

This study was conducted in three academic institutions, namely: St. John's University, Dodoma University College of social sciences, and the College of Business Education (CBE) Dodoma campus. All these three institutions are located in the Dodoma Municipal Council. St John's University registers health sciences, business, and education students at degree and master's levels. Dodoma University-College of social sciences registers students in programs on humanities and social sciences at degree and master's degrees, mainly based on business education programs.

### Study population

The targeted study population for this study was female undergraduate students.

### Sampling technique

#### Sample size determination

The study involved 470 participants, a sample size calculated using Cochran's formula [11].

$$n=\frac{{\mathrm{Z}}^{2}\mathrm{pq}}{{\mathrm{e}}^{2}}$$

where n is the sample size, z is the value for 95% confidence interval = 1.96 on a normal distribution, q is the value of 1-p, e is the desired level of precision/marginal error = 0.05, and p which is the proportion of the population with the characteristic of interest. The value of p in this study was 50% because no previous research has documented Metronidazole use as an emergency contraceptive.

$$n=\frac{1.9{6}^{2}\times 0.5\times 0.5}{0.0{5}^{2}}$$

$$n=384$$

The sample size of this study was 384 + 10% non-response making a minimum of 422 participants.

### Sampling procedure

The stratified random sampling technique was used to obtain a sample size of 470. The selected 3 institutions (strata) register students taking different programs; St. John's University -health and education, Dodoma University College of social sciences – social sciences, and CBE – business education. From each stratum, a minimum of 30% of the required number of participants was expected and from each institution, random sampling was then employed until the agreed sample size was achieved.

### Data collection technique

Data for this study were collected by using a self-administered English-structured questionnaire. We collected data from one academic institution to another until the sample size of 470 was achieved. We met the participants in person during break time, explained the aim of the study, and eligible participants signed the consent form. Participants were then given the questionnaire to fill out and submit immediately. The filled questionnaires were collected and crosschecked for any missing information.

### Questionnaire

English structured questionnaire was used to collect data for this study. This tool was developed based on the study objectives since there is currently no published tool or previous study on this topic. The instrument comprised twenty questions; part I: sociodemographic characteristics and part II (information on Metronidazole and contraceptives use in multiple choice).

### Validity and reliability

The tool was shared with pharmacy research experts to assess for content validity, and no significant comments were raised, rather grammatical and arrangement of the questions. All comments were addressed before the final tool was ready for pretesting. Additionally, the tool was pretested on 20 participants to get feedback on user-friendliness and clarity, and positive feedback was received. Moreover, the collected information was analyzed to determine the internal consistency of the tool that revealed acceptable results, Cronbach alpha 0.847.

### Data analysis

Data collected were analyzed using the SPSS version 25. Data was carefully entered and crosschecked, and cleaned before actual analysis. Descriptive statistical analysis was done to summarize the demographic data and information on Metronidazole or other contraceptive use. A Chi-square test was done to determine the association between demographic information and Metronidazole use as medicine for emergency contraception *p* < *0.05* considered to be statistically significant. Multivariate binary logistic regression was further done to determine the nature of the association between the study variables.

## Results

### Sociodemographic characteristics of the participants

This study included a total of 470 participants with the majority 235(50%) at the age of 18–21 years. The majority 202(43%) of the participants were in the second year of study and single/informal relationships 446(94.9%) (Table 1).

**Table 1:** Socio-demographic characteristics of participants

Variable	Frequency (N)	Percentage (%)
**Age group**		
18–21	235	50
22–25	210	44.7
26–29	25	5.3
**University**		
University of Dodoma	138	29.4
St. John's University of Tanzania	187	39.8
College of Business Education	145	30.9
**Year of study**		
First-year	120	25.5
Second-year	202	43
Third-year	102	21.7
Fourth-year	46	9.8
**Marital status**		
Single/Informal relationship	446	94.9
Married	24	5.1

### Information on Metronidazole use and other contraceptives

We found that, among participants with experience using emergency contraceptives, 169(62.4%) used Metronidazole. Additionally, 345(73.4%) study participants said they had heard someone use Metronidazole for contraception, especially their peers (Table 2).

**Table 2:** Information on Metronidazole use and other contraceptives

Variable	Yes N (%)	No N (%)	Total N
Ever heard about emergency contraceptive methods	448 (95.3)	22(4.7)	470
P2	427(95.3)	21(4.7)	448
Metronidazole	153 (34.2)	295(65.8)	448
Other	12 (2.7)	436(97.3)	448
Ever used any emergency contraceptive methods	271(60.5)	177(39.5)	448
P2	139(51.3)	132(48.7)	271
Other	51(19)	220(81)	271
Used Metronidazole as an emergency contraceptive	169(62.4)	102(37.6)	271
Ever heard someone using Metronidazole as an emergency contraceptive	345(73.4)	125(26.6)	470

### Information on Metronidazole use and other contraceptives

In this study, among the participants who reported having used Metronidazole as an emergency contraceptive, 133(78.7%) used 3–6 tablets, and the majority 133(78.7%) had used 1–2 times. Additionally, 246(71.3%) of the participants who heard their peers using Metronidazole for contraception reported taking 3–6 tablets; while, 234(67.8%), stated that they took it once. Most participants who used Metronidazole as an emergency contraceptive got the medicine from the pharmacy 71(42%). More than half of the study participants, 264 (56.1%), knew that Metronidazole is an antibiotic. At the same time, it was interesting to find that 116(24.7%) of the individuals reported that it could be used as an emergency contraceptive (Table 3).

**Table 3:** Information on Metronidazole use and other contraceptives

Variable	Frequency (N)	Percentage (%)	
**Dosage** **Number of tablets you take**			
3–6	133	78.7	
7–10	36	21.3	
**Repetitive** **How many times have you used**			
1–2	133	78.7	
3–4	36	21.3	
**How many times do they use/dose frequency**			
1	234	67.8	
2	93	27	
3	18	5.2	
**Number of tablets they take**			
3–6	246	71.3	
7–10	99	28.7	
**Source I get Metronidazole from**			
Friends	29		17.2
Hospital	65		38.5
Pharmacy	71		42
The remaining dose prescribed for other health problems	4		2.3
**My friend gets Metronidazole from**			
Friends	9		2.6
Hospital	55		15.9
Pharmacy	276		80
The remaining dose prescribed for other health problems	5		1.5
**Awareness about the use of Metronidazole** **Use of Metronidazole**			
Antifungal	84		17.9
Antibacterial	264		56.1
Medicine to prevent unwanted pregnancy	116		24.7
Other	6		1.3

### Factors associated with the use of Metronidazole as emergency contraceptive

In this study, different factors/characteristics of the participants were found to associate with using Metronidazole as an emergency contraceptive. The university from which the participant came from that is medical or non-medical was significantly associated with Metronidazole use *p-value* < *0.01.* Additionally, the participant's year of study and having a peer who used was also significantly associated with Metronidazole use as an emergency contraceptive *p* < *0.01* consecutively (See Table 4). Further analysis revealed that as the students advanced to the next year of study this was significantly associated with reduced use of Metronidazole as an emergency contraceptive (B = [-0.45], *p* = [0.02]). Additionally, although not statistically significant, being married and hearing about emergency contraceptives were negatively associated with using Metronidazole as an emergency contraceptive. Furthermore, an increase in age, studying in non-medical college/university, the experience of using contraceptive methods, and hearing someone ever used Metronidazole was found to be positively associated with Metronidazole use as an emergency contraceptive. Nevertheless, not statistically significant (Table 5).

**Table 4:** Association between participant characteristics and Metronidazole use for contraception

	Used Metronidazole as an emergency contraceptive		
Variable	Yes	No	*p*-value
**Age group**			0.21
18–21	71(68.9)	32(31.1)	
22–25	90(58.1)	65(41.9)	
26–29	8(61.5)	5(38.5)	
**Distribution of respondents per three Universities** **University**			0.01*
University of Dodoma	74(70.5)	31(29.5)	
St. John's University of Tanzania	46(41.8)	64(58.2)	
College of Business Education	49(87.5)	7(12.5)	
**Distribution respondents per study year** **Year of study**			0.01*
First year	31(59.6)	21(40.4)	
Second year	89(71.8)	35(28.2)	
Third year	46(68.7)	21(31.3)	
Fourth year	3(10.7)	25(89.3)	
**Distribution of respondents per marital status** **Marital status**			0.537
Single	159(62.8)	94(37.2)	
Married	10(55.6)	8(44.4)	
**Information on EC & Use of Metro** **Ever heard about emergency contraceptive methods**			0.717
No	1(50)	1(50)	
Yes	168(62.5)	101(37.5)	
**Heard of someone using Metronidazole as an emergency contraceptive**			0.01*
No	1(2.9)	34(97.1)	
Yes	169(71.3)	67(28.7)	

^*^p-value < *0.01*

**Table 5:** Multivariate Binary logistic regression

Variable	B	S.E	Wald	df	Sig	Exp(B)
Age group	0.28	0.31	0.87	1	0.35	1.34
University	0.12	0.19	0.41	1	0.52	1.13
Year of study	-0.45	0.19	5.34	1	***0.02***	0.64
Marital status	-0.27	0.61	0.20	1	0.66	0.76
Ever heard about emergency contraceptive methods	-0.09	1.93	0.00	1	0.96	0.91
Ever used any emergency contraceptive methods	2.15	1.23	3.05	1	0.08	8.66
Heard of someone using metronidazole to prevent pregnancy	6.79	5.66	0.00	1	1	16.91

### Reasons for using Metronidazole as an emergency contraceptive

**Fig. 1:**
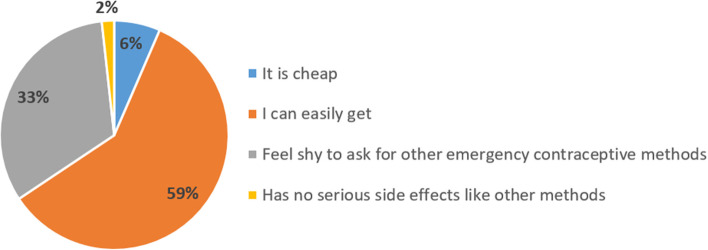
Reasons for using Metronidazole as an emergency contraceptive

We found that participants with experience of using Metronidazole as an emergency contraceptive had different reasons with the majority 100 (59%) reporting that it is easily accessible (Fig. 1).

## Discussion

This study found that most participants had heard about emergency contraceptives, similar to previous research on adolescents [12]. Although most participants knew P2 is an emergency contraceptive as supported by a previous study on contraceptive use and awareness [13], it was very interesting to find that more than 30% of the study participants had heard that Metronidazole could be used as an emergency contraceptive. This may be explained by a previous study among youths which revealed that the majority of them had awareness about contraceptives, although still some factors were found to affect their use of contraceptive services [12].

Also, some awareness gaps were revealed where some of the participants reported that they use other methods, such as condoms and pills, which are methods for preventing pregnancy but not emergency contraceptives. In addition, in the current study, more than half of the participants had the experience of using emergency contraceptives, especially P2. It was astonishing to find that more than 60% of the participants who have used contraceptives had the experience of using Metronidazole. This finding was interesting and can be explained by another study that linked antimicrobial misuse with social norms [14].

Additionally, among the participants who reported using Metronidazole as an emergency contraceptive, the majority used more than three tablets, mostly six tablets (1200 mg), and had used between 1–2 times. This oral use of metronidazole is very high and against the recommended adult prescription of 400 mg thrice a day for 5-7days [15]. This finding can be related to a report from a previous study that revealed that youths believed that high doses of drugs [9] could prevent pregnancy. Moreover, a previous study reported spermicidal activity of metronidazole vaginal gel after 1 h of 1gm application [9] which might support the reported use by adolescents in this study.

Furthermore, participants with experience using Metronidazole as an emergency contraceptive said that they got it from the pharmacy and the hospitals but were prescribed for other conditions. This finding was similar to a previous study which reported malpractices in antibiotic use such as self-medication with cocktails due to easy access to prescription medicines from pharmacies and unofficial suppliers [14]. Possibly, the availability of metronidazole, without legal prescription and failure to complete prescribed doses, explains the high percentage of participants obtaining the medicine and its unofficial uses. Moreover, it was exciting from the findings of this study that although more than half of the participants knew that Metronidazole was an antibiotic, some still reported it is an emergency contraceptive medicine.

The study found different characteristics of the study participants to associate with using Metronidazole as an emergency contraceptive. The university from which the study participant came was found to be significantly associated with Metronidazole use. Few participants from institutions providing medical programs used Metronidazole as contraceptive medicine compared to those from non-medical institutions. This may be explained by the fact that participants from medical institutions might have more awareness about pregnancy prevention and contraception and the possible consequences of using Metronidazole in high doses and for unofficial indication.

Also, as the participants advanced the year of study, this was negatively associated with the use of Metronidazole as an emergency contraceptive. This finding may be explained by a previous study by Bergström et al. which revealed that the class of adolescents significantly increased their knowledge and as their age increases, they get more exposure and knowledge about sex, pregnancy, and preventive measures [16].

Moreover, having peers with experience using Metronidazole as an emergency contraceptive was also found to associate with its use among the study participants. Previous studies also reported that using and misusing contraceptives and antibiotics have been associated with social life and peer influence [12, 14, 17–21]. This observation may be explained by the fact that youths and age mates can easily share information and influence each other resulting in participation in healthy or non-healthy behaviors.

In the present study, many reasons were identified to influence the use of Metronidazole as an emergency contraceptive. The majority of the participants reported that they used Metronidazole because it is easy to get compared to other methods similar to a previous study on antibiotic misuse [14]. Other reasons included being shy to ask for emergency contraceptive methods, being cheap, and feeling that it has no severe side effects. Previous studies on misuse of antimicrobials and contraceptives also mentioned similar reasons, such as myths and misconceptions on conception, lack of awareness and young people's preferred source of contraceptives, and easy access and availability of medicines without prescription [12, 17, 19, 20, 22].

Another study done in Nepal reported that lack of knowledge by the consumers and financial difficulties might be other reasons for antibiotic misuse [18]. In the present study, possibly due to the norms and culture and the fact that the majority are not in official relationships, these youths may experience difficulties accessing recommended emergency contraception, hence using any methods that are easily accessible and affordable.

### Limitations of the study

This study used a stratified random sampling technique to obtain the participants; selecting participants randomly from the strata might result in selection bias but we recruited a large sample considering different participants’ characteristics to get representativeness.

## Conclusion

This study revealed that Metronidazole was unofficially used as an emergency contraceptive in high doses apart from the therapeutic uses. This situation calls for interventions from different stakeholders such as the Ministry of Health, training institutions, regulatory authorities, and the general population to ensure that youths in all environments are well-informed and have access to safe methods of emergency contraception. Moreover, further research may be done on the toxic effects of metronidazole when used in high doses as emergency contraceptive and a comparison of efficiency between metronidazole and other emergency contraceptives.

## Data Availability

When necessary, the research tools, dataset, and other materials supporting the results will be shared upon consultation with the corresponding author.
